# TR4 nuclear receptor enhances prostate cancer initiation via altering the stem cell population and EMT signals in the PPARG-deleted prostate cells

**DOI:** 10.18632/oncoscience.121

**Published:** 2015-02-09

**Authors:** Shin-Jen Lin, Dong-Rong Yang, Nancy Wang, Ming Jiang, Hiroshi Miyamoto, Gonghui Li, Chawnshang Chang

**Affiliations:** ^1^ George Whipple Lab for Cancer Research, Departments of Pathology, Urology, Radiation Oncology, and The Wilmot Cancer Center, University of Rochester Medical Center, Rochester, NY, USA; ^2^ Department of Urology, the Second Affiliated Hospital of Suzhou University, Suzhou, China; ^3^ Department of Urologic Surgery, Vanderbilt-Ingram Comprehensive Cancer Center, Vanderbilt University Medical Center, Nashville, Tennessee, USA; ^4^ Department of Urology, Sir Run Run Shaw Hospital, Zhejiang University, Hangzhou, China; ^5^ Sex Hormone Research Center, China Medical University/Hospital, Taichung, Taiwan

**Keywords:** Prostate cancer, TR4, PPARG

## Abstract

A recent report indicated that the TR4 nuclear receptor might suppress the prostate cancer (PCa) initiation via modulating the DNA damage/repair system. Knocking-out peroxisome proliferator-activated receptor gamma (PPARG), a nuclear receptor that shares similar ligands/activators with TR4, promoted PCa initiation. Here we found 9% of PCa patients have one allele of PPARG deletion. Results from *in vitro* cell lines and *in vivo* mouse model indicated that during PCa initiation TR4 roles might switch from suppressor to enhancer in prostate cells when PPARG was deleted or suppressed (by antagonist GW9662). Mechanism dissection found targeting TR4 in the absence of PPARG might alter the stem cell population and epithelial-mesenchymal transition (EMT) signals. Together, these results suggest that whether TR4 can enhance or suppress PCa initiation may depend on the availability of PPARG and future potential therapy via targeting PPARG to battle PPARG-related diseases may need to consider the potential side effects of TR4 switched roles during the PCa initiation.

## INTRODUCTION

Testicular nuclear receptor 4 (TR4) belongs to the nuclear receptor superfamily and was first cloned in 1994 (1). TR4 physiological functions are involved in metabolism, cancer development, fertility, bone diseases, cardiovascular diseases, etc (2-11). Peroxisome proliferator-activated receptor gamma (PPARG), another nuclear receptor, also has multiple functions mostly in metabolism, cancer development, cardiovascular diseases, bone diseases, etc (12-19).

TR4 and PPARG share many similarities yet also have distinct functions in some selective diseases. First, both *TR4* and *PPARG* genes were located in the chromosome 3p at regions 3p24 and 3p25, respectively (20). Second, they share the same ligands/activators, i.e. polyunsaturated fatty acids and thiazolidinediones (TZDs), which transactivate their downstream target genes (11, 21–23). Third, they bind to the similar Hormone-Response-Elements (HREs) sequences, i.e. two consecutive AGGTCA sequences with spacing of 0-5 nucleotides (direct repeat 0-5) (23). However, more and more evidences indicate that these 2 nuclear receptors can also function oppositely in some selective diseases. First, they exert differential effects on insulin sensitivity with PPARG increasing insulin sensitivity (24-26) vs TR4 decreasing insulin sensitivity (4). Second, PPARG suppresses atherosclerosis (17) while TR4 enhances atherosclerosis (11). Third, PPARG increases osteoporosis (19) while TR4 decreases osteoporosis (10). These contrasting results suggest that TR4 and PPARG may act as competitors for their upstream ligands and/or their downstream target gene modulation.

Lin et al recently reported that TR4 played a protective role in PCa initiation *via* modulation of the DNA damage/repair pathway (27). They showed TR4 could suppress PCa development in 3 different mouse models and 2 different cell lines. Interestingly, Jiang et al also reported similar results showing knocking-out PPARG resulted in enhanced prostatic intraepithelial neoplasia (PIN) in the mouse model (15). Here we report that TR4 can enhance or suppress PCa initiation depending on the availability of PPARG.

## RESULTS

### Selective PCa patients have one allele *PPARG* deletion

**Figure 1:**
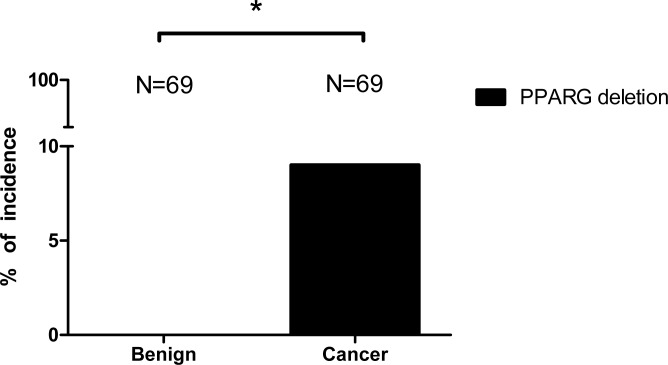
9% of PCa patients have PPARG deletion 69 PCa samples were tested by FISH. 6 out of 69 cancer specimens showed one allele PPARG deletion while none of the normal compartments of those 69 samples showed the PPARG deletion. The statistic results were calculated using Fisher's exact test. * P<0.05.

We examined PPARG deletion in PCa tissue microarray by FISH and found 9% (6 out of 69) of the PCa samples have one allele *PPARG* deletion (Fig. 1). In contrast, the control tissue microarray from the same patients’ normal/benign compartment has zero PPARG deletions (Fig. 1). Together, results from human clinical sample surveys suggested that PPARG deletion might be linked to the PCa development.

### TR4 increases normal prostate epithelial PPARG-deleted cell growth and transformation

**Figure 2:**
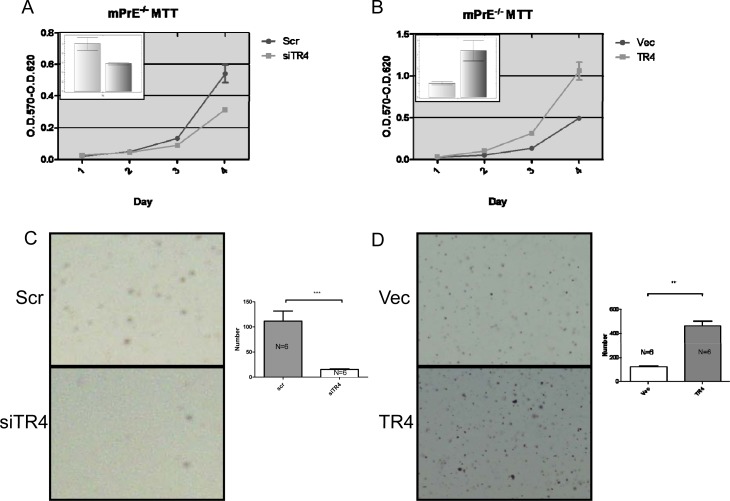
TR4 increases PPARG null normal prostate epithelial cell growth and transformation (A-B) Cell growth was assayed by MTT in the PPARG null normal prostate epithelial cell line mPrE^−/−^. TR4 knockdown (siTR4) compared to scramble control (Scr) shown in (A) and TR4 over-expressed (TR4) compared to vector control (Vec) shown in (B). The inset graphs represent the TR4 mRNA level by QPCR. (C-D) Anchorage independent cell growth was assayed by agarose colony formation. TR4 knockdown compared to scramble control shown in (C) and TR4 over-expressed compared to vector control shown in (D). Right panels show the quantification of the colony numbers. **P<0.01, ***P< 0.001

An early report suggested that PPARG had high homology with TR4 and shared similar ligands/activators (23), we were interested to see the potential differential effects of TR4 on PCa development in the normal prostate cells with or without PPARG deletion. We first applied the normal prostate epithelial mPrE^−/−^ cell line cloned from the PPARG knockout mouse to study the TR4 effects on the PCa development. Using TR4-siRNA or TR4-cDNA to manipulate the TR4 expression, followed by the carcinogen NMU treatment to induce cell transformation (27, 28), we found knocking-down TR4 suppressed mPrE^−/−^ cell growth using MTT assay (Fig. 2A). In contrast, addition of TR4 led to enhance mPrE^−/−^ cell growth (Fig. 2B). Importantly, we also found knocking-down TR4 suppressed mPrE^−/−^ cell transformation in the presence of carcinogen NMU (Fig. 2C), and addition of TR4 led to enhance mPrE^−/−^ cell transformation using anchorage independent colony formation assay (Fig. 2D).

Interestingly, instead of knockingout PPARG, we applied the PPARG specific antagonist GW9662 (29) to suppress PPARG function, and results revealed that knocking-down TR4 led to suppress PPARG intact cell line mPrE^+/+^ cells treated with GW9662 (Fig. S1A). Similar results were also obtained when we replaced mPrE^+/+^ cells with normal human prostate epithelial RWPE1 cells (Fig. S1B).

Together, results from Fig. 2 and S1 indicated that in the prostate cells with deleted or reduced PPARG, knocking-down TR4 could suppress prostate cell growth and transformation.

### TR4 enhances the PPARG-deleted prostate tumor development in the *in vivo* mouse model

**Figure 3:**
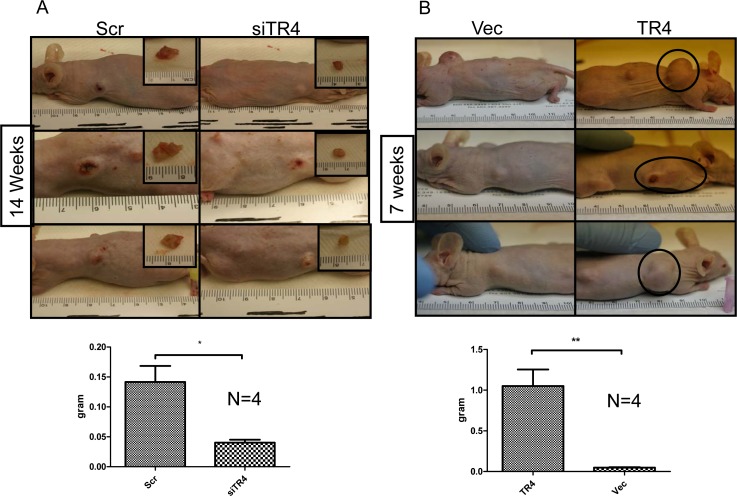
TR4 increases PPARG null normal prostate epithelial cell tumor formation 10^6^ of mPrE^−/−^ cells were inoculated subcutaneously in nude mice (N=4 in each group). (A) TR4 knocked-down mPrE^−/−^ cells (siTR4) were inoculated into the left side compared to the vector control inoculated into the right side (Scr). The mice were sacrificed at 14 weeks. The inset pictures represent the tumors removed from the mice. The quantification data of tumor weights were shown in the bottom panel. P-values < 0.05 (*) were calculated by Student's t test. (B) TR4 over-expressed mPrE^−/−^ cells (TR4) were inoculated into the right side compared to the vector control inoculated into the left side (Vec). The mice were sacrificed at 7 weeks. The black circles represent the xenograft tumors. The quantification data of tumor weights were shown in the bottom panel. P-values < 0.01 (**) were calculated by Student's t test.

To confirm those *in vitro* studies showing TR4 enhanced prostate epithelial mPrE^−/−^ cell growth and transformation in *in vivo* mouse model, we prepared xenografts of mPrE^−/−^ cells with manipulated TR4 expression in nude mice. Results from Fig. 3A indicated that knocking-down TR4 led to reduced tumor sizes while over-expression of TR4 led to increased tumor sizes. Mice with TR4 knocked-down cells were sacrificed at 14 weeks after xenografts and mice with TR4 over-expressed cells were sacrificed at 7 weeks, suggesting the tumor growth was much faster in the mPrE^−/−^ TR4 over-expressed group (Fig. 3B).

Results from Fig. 3 confirmed the above *in vitro* studies and concluded that TR4 might play a positive role in the PCa initiation when PPARG is deleted in prostate cells.

### TR4 enhances prostate trans-differentiation in PPARG-deleted prostate cells

**Figure 4:**
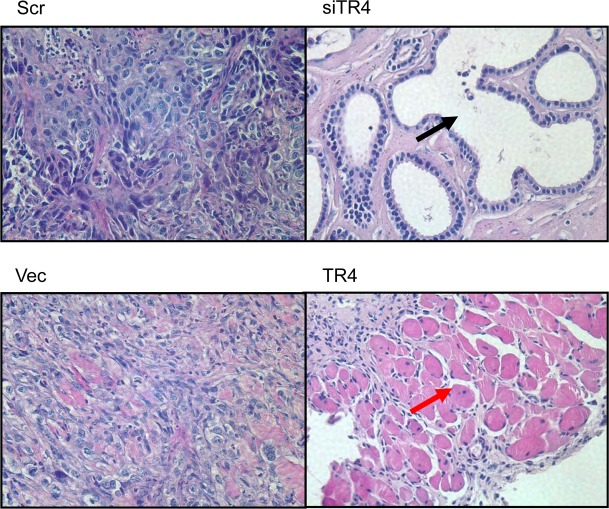
TR4 increases prostate trans-differentiation in PPARG null normal prostate epithelial cell The tumors from the xenografted nude mice in Figure 3 were collected and processed. H&E staining were performed in the scramble control (Scr), TR4 knockdown (siTR4), vector control (Vec), and TR4 over-expressed (TR4) tumor samples. The black arrow indicates the luminal structure and the red arrow indicates the muscular structure.

Interestingly, H&E staining of the xenografted PCa tissues prepared for Fig. 3 also found that tumors retained the adenocarcinoma structure when TR4 was knocked-down (Fig. 4). However, we found that tumors might develop into muscular structure when TR4 was over-expressed (Fig. 4). These contrasting results suggest that PCa may undergo the trans-differentiation process from the epithelial type to the mesenchymal type of cancer after TR4 was altered in the PPARG-deleted prostate cells.

### TR4 increases stem cell population in PPARG-deleted prostate cells

There are several ways to trans-differentiate the epithelial cells into the mesenchymal cells (30-34). The first way is to de-differentiate epithelial cells back into stem cells, and the resulting stem cells could then differentiate into many types of cells depending on the environment (30-32).

**Figure 5:**
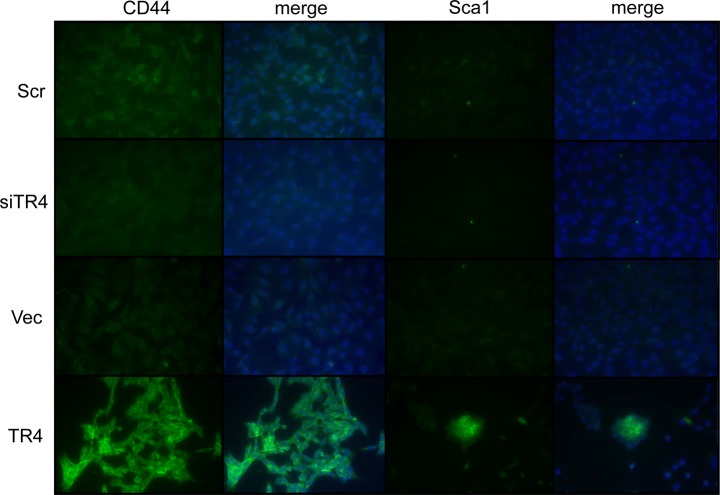
TR4 increases stem cell population in PPARG null normal prostate epithelial cell Scramble control (Scr), TR4 knockdown (siTR4), vector control (Vec), and TR4 over-expressed (TR4) mPrE^−/−^ cell lines were stained by CD44 and Sca1 antibodies. The right panels next to CD44 or Sca1 show the images merged with DAPI staining.

We first tested the stem cell marker CD44 and found knocking-down TR4 decreased CD44 expression while over-expressing TR4 significantly enhanced the CD44 expression in mPrE^−/−^ cells. Similar results also occurred when we examined another stem cell marker, Sca1 (Fig. 5).

Together, results from Fig. 4-5 suggest that TR4 may be able to increase the prostate cells de-differentiation into the stem cells in the PPARG-deleted cells; then in the *in vivo* xenografted environment, cells can differentiate into either epithelial or mesenchymal type of tumors.

### TR4 increases EMT in PPARG-deleted prostate cells

**Figure 6:**
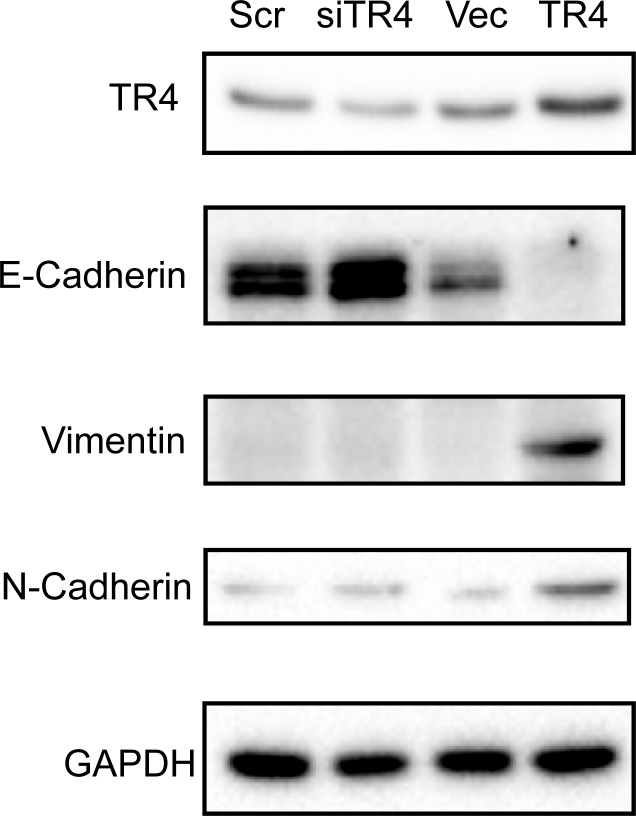
TR4 increases EMT in PPARG null normal prostate epithelial cell mPrE^−/−^ cell lines with TR4 knockdown (siTR4) or over-expressed TR4 (TR4) were assayed for TR4 and the EMT related proteins E-Cadherin, Vimentin, and N-Cadherin expression by Western Blot.

Another way to turn the epithelial cells into the mesenchymal cells is through the epithelial-mesenchymal transition (EMT) (33, 34). We extracted proteins from the mPrE^−/−^ cell lines to investigate the EMT marker levels. We found E-Cadherin protein increased in the TR4 knocked-down mPrE^−/−^ cells, while it was almost undetectable in the TR4 over-expressed mPrE^−/−^ cells, indicating the strong EMT phenotype (Fig. 6). We further confirmed this EMT *via* testing other markers and found Vimentin and N-Cadherin were also increased in the TR4 over-expressed mPrE^−/−^ cells (Fig. 6).

Together, results from Figs. 4-6 suggest that TR4 may play key roles to promote the trans-differentiation in the xenografted mice either *via* altering the stem cell transition and/or EMT in the PPARG-deleted cells.

## DISCUSSION

Here we provide the evidences that a nuclear receptor (TR4) impact on PCa progression can be switched based on the availability of another nuclear receptor (PPARG). This is not only unique among nuclear receptors, but it may also have an impact on the future drug development that is based on targeting the nuclear receptors. Early studies suggested that TR4 is a tumor suppressor through regulating the DNA damage/repair system to delay the PCa initiation (27). Results from current studies, however, demonstrate that TR4 can function as a tumor enhancer *via* regulating the stem cell population and EMT to promote PCa initiation if PPARG is deleted or its function is suppressed by the GW9662 inhibitor. Both cancer stem cell and/or EMT induced cancer initiation have been reported recently (35-38). Surprisingly, EMT also contributes to the benign prostatic hyperplasia (39), indicating EMT is not only essential in the later metastatic stage (33, 34), but also plays critical roles in the cell proliferation and cancer initiation.

Why can a single nuclear receptor have these contrasting roles? While the potential explanation could be due to a simple competition between these 2 receptors, the exact mechanism is still elusive and needs further detailed investigations. For instance, under the normal PPARG circumstances, TR4 may function as a tumor suppressor to repair the damaged DNA with little ability to change the stem cell population and/or EMT. However, under the PPARG deleted circumstances (either the gene is lost or loses function), TR4 may still keep its DNA damage repair function but also gain the ability to increase the stem cell population and ability to alter the EMT.

Future clear mechanism dissection of how these 2 receptors interact would resolve the puzzles why TR4 and PPARG have those opposite roles in different diseases including insulin resistance, atherosclerosis, osteoporosis, and now PCa. Importantly, future drugs *via* targeting either TR4 or PPARG may need extra consideration and be re-examined carefully for additional potential side effects. For example, the anti-diabetic drug TZDs may enhance both PPARG and TR4 activities. However, if the patients have the one allele PPARG deletion, then taking TZD may have the potential risk to promote the PCa development. A better approach will be to examine the PCa patients’ TR4 and PPARG expression in advance before receiving any drugs based on targeting either of these 2 receptors.

## MATERIALS AND METHODS

### Prostate cancer and paired benign prostate samples

69 cases of human PCa and paired benign appearing prostate specimens were provided by the Surgical Pathology archives after appropriate approval from the Institutional Review Board of the University of Rochester Medical Center.

### Fluorescence In situ Hybridization (FISH)

In situ hybridization was performed by the University of Rochester Medical Center Pathology Lab. Green 5-Fluorescein dUTP labeled Human BAC Clone RPCI-11 109P2 probes for human *PPARG* were purchased from Empire Genomics and we followed their protocol for hybridization.

### Cell culture

The spontaneously immortalized mouse prostatic epithelial cell lines, mPrE^−/−^ or mPrE^+/+^, were generated by Dr. Min Jiang (15) from the *PPARG* knockout or wild type mouse, respectively. They were maintained in RPMI 1640 media (GIBCO) supplemented with 10% fetal bovine serum and 1% Antibiotic-Antimycotic solution (Invitrogen). The RWPE1 cell line was obtained from the American Type Culture collection (ATCC, Rockwell, MD) and maintained in complete keratinocyte serum-free media (KSF media), supplemented with 1% penicillin/streptomycin/amphoterycin B, 50 mg/ml bovine pituitary extract and 5 ng/ml epidermal growth factor (Life Technologies, Barcelona, Spain). Stable cell lines expressing scramble shRNA (scr) or shRNA (CGGGAGAAACCAAGCAATT) against TR4 (TR4-siRNA) were established by transfecting pcDNA6/TR and pSuperior.retro.puro plasmids into mPrE^−/−^, mPrE^+/+^, or RWPE1 cells and selected for stable cell lines by treatment with blasticidin (12 μg/ml) and puromycin (1.2 μg/ml) for two weeks. Tetracycline (1 μg/ml) was added in order to induce shRNA expression. Stable cell lines expressing TR4 or vector control were established by transfecting pcDNA5/TO-TR4 (TR4-cDNA) or pcDNA5/TO (Vec) together with pcDNA6/TR plasmids into mPrE^−/−^ cells and selected for stable cell lines by treatment with blasticidin (12 μg/ml) and hygromycin (100 μg/ml) for two weeks. Tetracycline (1 μg/ml) was added in order to induce TR4 expression.

### RNA extraction and quantitative PCR

RNA was extracted from cell lines using TRIzol® reagent (Invitrogen) and converted to cDNA by Superscript III transcriptase (Invitrogen). QPCR was performed using Bio-Rad CFX96 system with SYBR green (Bio-Rad) to determine the level of mRNA expression of a gene of interest. Expression levels were normalized to the expression of β-actin RNA.

### Cell proliferation assay

Cells were sub-cultured to 10% confluence then treated with 100 μM N-nitrosomethylurea (NMU) or vehicle control DMSO for 2 hours. Then the cells were grown to reach 100% confluence. Repeat the procedure of NMU treatment for total 3 cycles. After 3 cycles of treatment, passage the cells 5 more times. The treated mPrE^−/−^, mPrE^+/+^, or RWPE1 cells were then seeded in 24-well plates (3000 cells/well) and cultured for 24, 48, 72, or 96 hours. After each time point, cell numbers were calculated after using MTT assay staining.

### Cell transformation/colony forming assay

Cells were treated with 100 μM NMU or vehicle control DMSO for 3 cycles followed by 5 passages. Cells were suspended at a density of 2×105 cells/ml in 0.4% Noble agar (Sigma, St. Louis, MO) containing media and seeded on top of 0.8% agarose containing media in culture plates plus 2 ml media on top of agar. Media were changed every week for one month. Colonies were stained with p-iodonitrotetrazolium violet (Sigma, St. Louis, MO), photographed, and counted.

### Subcutaneous xenograft model

Male 6-8 weeks old nude mice were purchased from NCI. 1×106 cells with matrigel mixture were subcutaneously injected into the nude mice at 8 weeks of age. The tumors were evaluated at 7 or 14 weeks after injection.

### Tissue processing and H&E Staining

Xenografted tumor tissues were collected and fixed by 10% formalin followed by paraffin embedding. Samples were sliced to 5 μm thickness followed by hematoxylin and eosin staining.

### Cell immunofluorescence staining

105 cells were seeded in 4-well chamber slides. After 24 hours, cells were fixed with acetone for 5 minutes. After 30 minutes blocking, 2 μg/ml of primary antibodies anti-CD44 or anti-Sca1 conjugated with fluorescence were added to the slides overnight at 4°C. Cells were then mounted with mounting solution containing DAPI nucleus staining and pictures were taken with fluorescence microscopy.

### Western blot analysis

Target cells were harvested and lysed in RIPA buffer (50 mM Tris–HCl/pH 7.6: 150 mM NaCl: 1% Triton-x-100: 1% sodium deoxycholate: 0.1%SDS) supplemented with complete mini protease inhibitor cocktail tablets. Protein concentration was estimated using the Bio-Rad protein assay (Bio-Rad, Marnes-la-Coquette, France). 50 μg of sample proteins were loaded and run on the 10% SDS-PAGE gel and transferred to PVDF membranes (Millipore). Nonspecific binding was blocked in TBST buffer (0.5 mM Tris-HCl, 45 mM NaCl and 0.05% Tween 20, pH 7.4) containing 5% non-fat powdered milk. Membranes were incubated with primary antibodies anti-E-Cadherin (R&D), anti-Vimentin (Santa Cruz), and anti-N-Cadherin (Abcam) overnight at 4°C. Membranes were then washed in TBST and incubated with HRP conjugated second antibody for 1 hr. The blots were then washed in TBST, followed by incubating with ECL solution according to the manufacturer's instructions (GE Healthcare).

### Statistics

The data values were presented as the mean±SEM. P values were calculated by unpaired Student's *t* test or Fisher's exact test. P < 0.05 was considered statistically significant.

## SUPPLEMENTARY MATERIAL FIGURE


