# Supplementary material for: TR4 nuclear receptor enhances prostate cancer initiation via altering the stem cell population and EMT signals in the PPARG-deleted prostate cells
Source: Oncoscience. 2015 Feb 9;2(2):142–50. doi: 10.18632/oncoscience.121 (PMC4381707; doi:10.18632/oncoscience.121)
Supplement: Supplementary file 1 [file oncoscience-02-0142-s001.pdf]

TR4 nuclear receptor enhances prostate cancer initiation via altering the stem cell population and EMT signals in the PPARG-deleted prostate cells

Supplementary Material

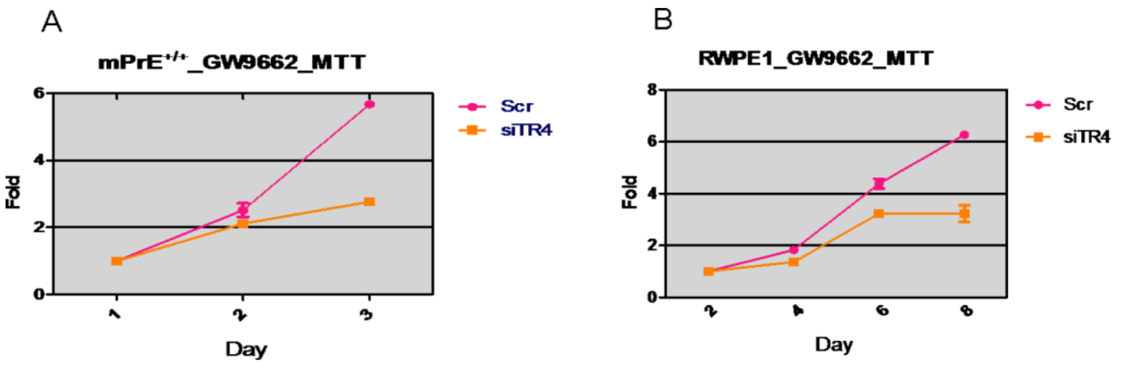

Figure S1: Knockdown TR4 suppresses mPrE$^{+/+}$ & RWPE1 cell growth following the PPARG specific antagonist treatment.
